# Comparison of the Isometric Position of the External Elbow Fixator: Self-Centering Versus Traditional Techniques, Postoperative CT Evaluation, and 3D Motion Analysis

**DOI:** 10.3390/jcm14113653

**Published:** 2025-05-23

**Authors:** Prospero Bigazzi, Chiara Suardi, Anna Rosa Rizzo, Irene Felici, Marco Biondi, Andrea Poggetti, Sandra Pfanner

**Affiliations:** Hand and Reconstructive Microsurgery Unit, AOU Careggi, 50134 Florence, Italy; pbigazzi@prosperius.it (P.B.); annar.rizzo@gmail.com (A.R.R.); irenefelici1@gmail.com (I.F.); marcobiondi@ymail.com (M.B.); poggetti.andrea@gmail.com (A.P.); pfanners@aou-careggi.toscana.it (S.P.)

**Keywords:** elbow external fixator, self-centering, elbow center of rotation, elbow fracture, elbow ligament reconstruction

## Abstract

**Background/Objectives**: The external hinged elbow fixator is a surgical choice both in the case of simple dislocations and elbow dislocation fractures. The correct positioning with respect to the elbow’s center of rotation is demanding. Authors developed a self-centering external fixator that does not require a pin in the elbow’s center of rotation. The aim of this study was to analyze the margin of error in its positioning. **Methods:** We subjected 16 patients to a CT-3D study reconstruction using 3D motion software to analyze the divergence angle and offset between the elbow’s center of rotation and that of the external fixator. The results were compared to those published on traditional implants. **Results:** All elbows were correctly reduced without re-dislocation. The average distance was 2° in relation to the center of rotation in the antero-posterior view, 3° in the cranio-caudal, and 2° in the medio-lateral. The divergence angle was 3.5° (min 0.4°; max 9.3°) and the offset 6.8 mm (min 0.06; max 17.5). The average range of motion was 10–145 (range 0–155). **Discussion:** The traditional hinged elbow external fixator creates severe complexity for surgeons in the necessary positioning of the elbow axial rod to correctly align the implant. The self-centering device avoids this step, making the procedure faster and easier. Although the alignment is still not perfect, the results are still comparable with traditional devices. **Conclusions:** The self-centering external fixator allows for correct alignment with the elbow’s center of rotation. It is less invasive and simpler, with a shorter learning curve, faster operating time, and less radiographic exposure.

## 1. Introduction

Hinged elbow external fixators have the advantage of simultaneously maintaining the concentric reduction in the ulnohumeral joint and allowing for early postoperative movements in one or more planes, neutralizing the external forces on soft tissues and bones [1,2]. Indications include both simple or complex acute fracture dislocation and chronic dislocation or stiffness elbow [3,4,5]. They can be used in association with procedures such as distraction interposition arthroplasty, ligament reconstruction, and contracture release [6,7]. In the unstable elbow, the principle is to provide stability as a static external fixator, allowing elbow flexion–extension in the early postoperative period. Conventional hinged elbow external fixators require, for a correct assembly, a provisional rod to be inserted into the center of rotation of the elbow, usually through a lateral approach [8]. This results in a challenging time-consuming procedure with high radiological exposure.

In 2015, the authors developed an auto-centering hinged external fixator to eliminate the surgically challenging and time-consuming step of inserting the provisional rod into the elbow’s center of rotation [1]. The aim was to make an easier-to-position external fixator with a self-centering mechanism. From the first prototype of 2015, we then modified the construct to make it lighter, make it simpler to use, and allow for 30° varus–valgus correction. The final device, Clickit Er Elbow^®^ (MIKAI S.P.A., Genova, Italy), obtained European Certification (CE) and has been produced and distributed by Mikai S.p.A.

The primary aim of this study was to analyze whether the self-centering external fixator was accurate or not in determining the position of the elbow’s center of rotation. For this purpose, we first subjected all patients in the immediate postoperative period to a TC study of the elbow, including the external fixator. We then transferred the data to a 3D motion software (Osirix vers 12.5) to identify the two axes: one passing through the anatomical center of rotation of the elbow and the other through the center of rotation of the device, as previously reported in a study of Soubeyrand [9]. We then recorded the divergences between them in terms of the offset and divergence angle to analyze the real position of the hinge in relation to the anatomical elbow’s center of rotation.

In the field of traumatology, the use of 3D models with CT reconstructions is an important aid, especially in cases of fractures with complex joint involvement. In deformed limbs, it sometimes may not be possible to restore the correct anatomy without 3D reconstruction templates [10].

Our results have been compared to those reported in the literature [9] to verify the hypothesis that the self-centering external fixator is correctly aligned to the same extent as external fixators. We also registered the follow-up as clinical outcomes.

## 2. Materials and Methods

From 2011 to 2021, 16 hinged auto-centering external fixators were applied. We retrospectively evaluated each of them. The inclusion criteria were acute and subacute fracture dislocation, simple dislocation, post-traumatic instability of the elbow at every age, and a minimum follow-up (FU) of 12 months. Details about the surgical procedure with bone and ligament repairs/reconstruction are summarized (Table 1). Every patient gave informed consent to participate in the study.

The surgical technique can also be found on the technical brochure, Clickit Er Elbow^®^, Mikai.

**Figure 1:**
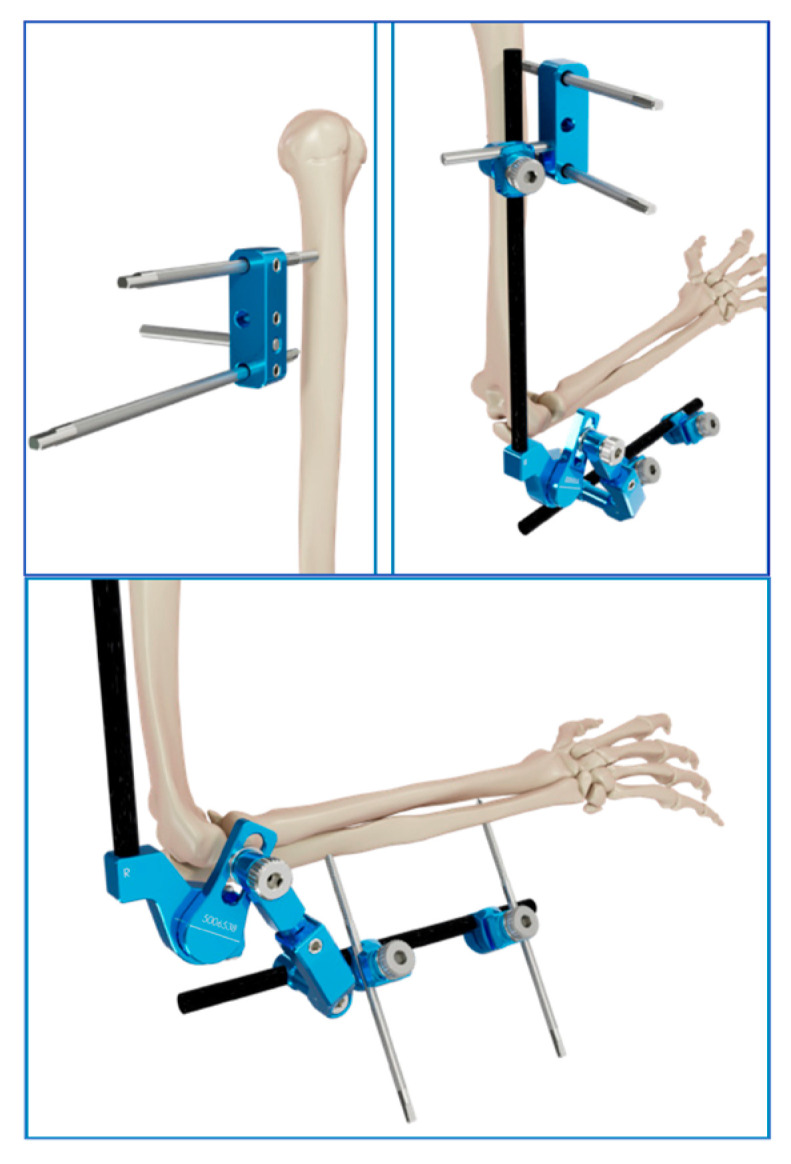
External fixator rod’s positioning.

**Figure 2:**
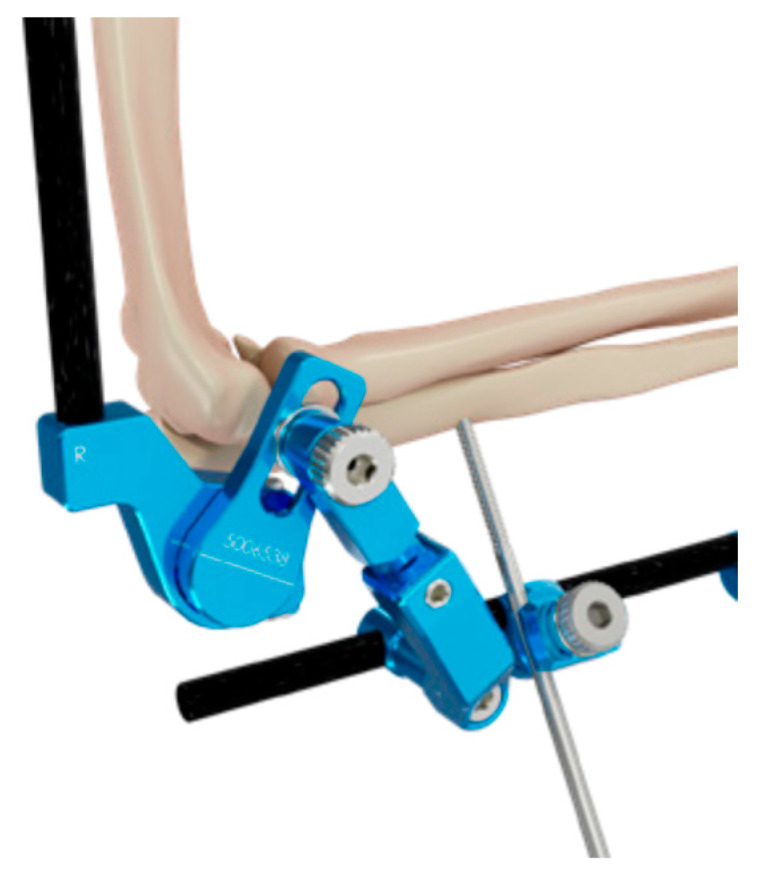
Details of the hinge.

**Figure 3:**
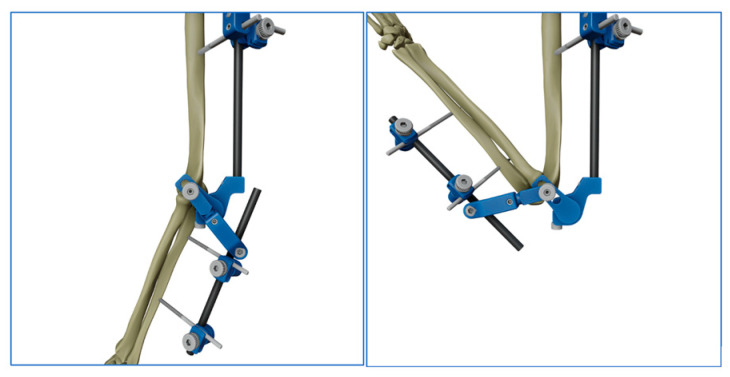
Iconographic representation of hinge lock when the elbow’s center of rotation is found.

**Figure 4:**
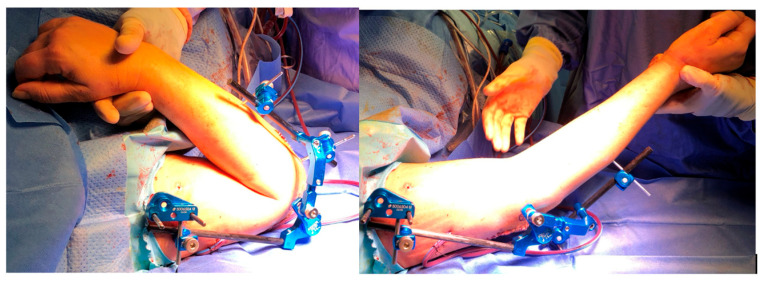
Clinical representation of hinge lock when the elbow’s center of rotation is found.

**Figure 5:**
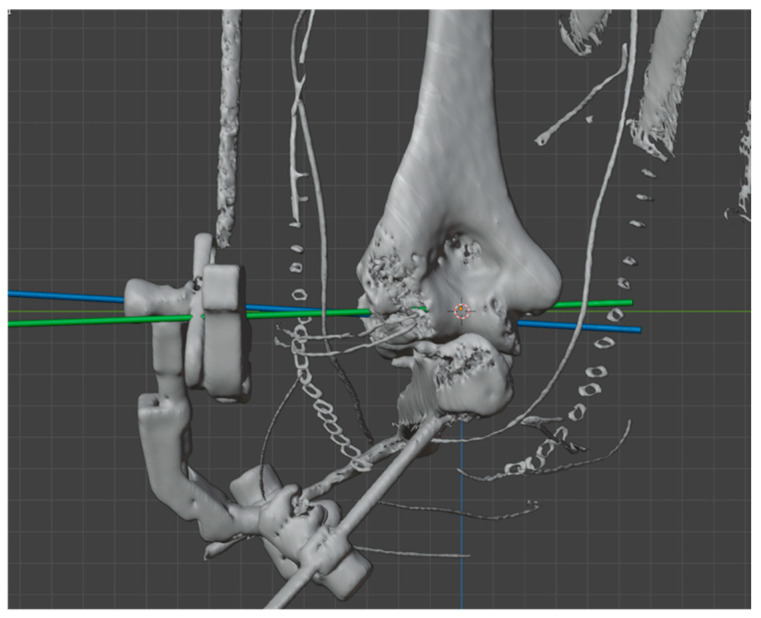
TC-3D study of the position of the hinge relative to the elbow’s center of rotation. Green line: elbow center of rotation. Blue line: external fixator center of rotation.

**Figure 6:**
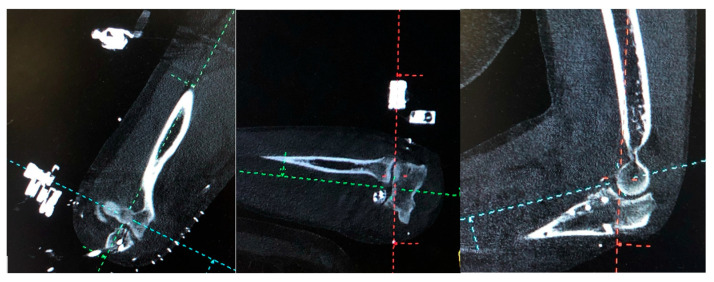
CT-3D in three projections with respect to the center of rotation of the elbow. Red line: external fixator center of rotation. Green line: elbow center of rotation.

The surgical procedure consists of a reduction in the humerus-ulnar joint, the position of 4 half-pins with small stab wounds, and two bicortical pins on the humerus, placed from lateral to medial positions and connected with clamps to the fixator humeral rod. The configuration could be with one pin placed close to the deltoid tuberosity and the other in the lower humeral shaft or with two proximal pins, close to the deltoid tuberosity, connected to a single clamp. The other 2 half-pins are placed posterolaterally from the posterolateral aspect of the ulna and connected with clamps to the fixator’s ulnar rod (Figure 1). After a gross visual alignment of the external fixator’s center of rotation with the elbow epicondyle, all the connections are fixed. The external fixator has a hinge that allows a range of freedom to find the axis elbow of rotation (Figure 2). With the holding elbow reduced, the fixator’s mechanical joint is then set free and allowed to align with the axis of elbow rotation during several repeated cyclic flexion–extension motions between 35° and 100°. As soon as the external fixator joint found its rest position, it was locked in, securely holding elbow reduction and allowing for flexion–extension movement (Figure 3 and Figure 4). After surgery, every patient was studied via a TC study to verify the position of the hinge relative to the center of rotation using a dedicated program (OsiriX MD 12.0). The radiological data have been transferred to this 3D motion software to identify the axis passing throw the anatomical center of rotation of the elbow and throw the center of rotation of the device (Figure 5 and Figure 6). The true center of rotation of the elbow was virtually materialized by using the following anatomical landmark: the tip of the lateral epicondyle and the line tangent to the inferior edge of the medial epicondyle, as reported in the literature [11]. The angle between the axis and the pin was measured (divergence angle) as well as the offset between both axes on the trochlea’s midline in millimeters. All the measurements have been performed by two external collaborators and the mean data recorded. A comparison was then made between our data and those previously observed from a cadaveric study with traditional external fixators [9].

In the immediate postoperative period (postoperative day 1), patients were instructed to mobilize in flexion–extension as far as they could tolerate based on pain. The external fixator was removed in the outpatient clinic in the standard way at 6 weeks post operation after X-rays were performed. The clinical outcomes were addressed as the range of motion (ROM) at latest follow-up and the absence of pain.

## 3. Results

**Figure 7:**
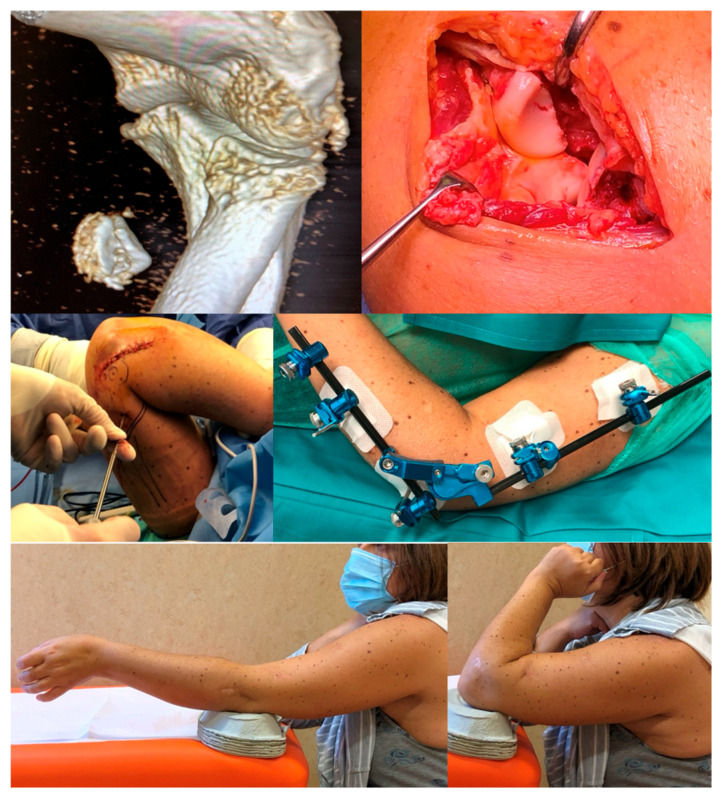
Clinical example of external fixator application and long-term follow-up.

The 3D-TC study showed a median error of the external fixator’s positioning of 2° in relation to the center of rotation in the antero-posterior direction, 3° in the cranio-caudal direction, and 2° in the medio-lateral direction. The average range of motion at the last follow-up was 10–145 (range 0–155) (Figure 7). All patients resumed their previous daily and work activities without pain. One patient underwent Total Elbow Arthroplasty due to pan arthrosis, while another sustained a coronoid resorption, needing a reconstruction with an allograft.

To analyze the hypothetical difference compared to the results of the traditional external fixators, we referred to a cadaveric study in which an analysis was made in terms of the angular divergence (in angular degrees) and angular divergence between the hinged external elbow fixator’s and flexion–extension’s axis [7]. In the study of Soubeyrand, six cadavers were studied by implanting a hinged external elbow fixator and two parameters (divergence angle and offset) were analyzed. Their results showed a median divergence angle of 4.45° and an offset of 4.1 mm. In the present study, the self-centering external fixator had a divergence angle of 3.5° and an offset of 6.8 mm. To analyze whether the results between were significantly different, a t-test was used. We performed the Kolmogorov–Smirnov test and verified the non-parametric distribution of the data. Then, chi-square tests were applied to compare the populations. Any results were considered statistically significant if *p* < 0.05. The p value was 0.3, and so not statistically significant. This represents a minimal difference, so the authors think that the external fixator with a self-centering technique cannot be considered either superior or inferior to the traditional technique, but comparable in terms of precision. The clinical outcomes of the patients are summarized in Table 1. Every patient was free of pain and resumed their previous activities.

## 4. Discussion

Elbow joint fracture dislocation occurs in a described predictable pattern. The primary stabilizers of the elbow are the anterior band of the medial collateral ligament, the ulnohumeral articulation, and the ulnar collateral ligament. The radial head is a secondary stabilizer that has a primary role when ligaments are torn [8]. It is stated that the best treatment of fracture dislocation of the elbow is to maintain anatomic joint congruence through a full arc of early flexion and extension [12,13].

The development of an articulated or hinged external fixator to treat unstable elbows has made possible maintaining the motion of the joint and permitting immediate postoperative exercise, preventing the hazardous translation of the elbow [14,15]. Hinge external fixators exist as uniplanar and multiplanar patterns. The uniplanar one allows the elbow to move in flexion–extension, constraining the varus–valgus and rotational movements. The multiplanar one offers control in multiple planes, but its application is more technically demanding and has a bulky structure, potentially causing skin problems [1].

Despite several hinged external fixators being available, they all share the same principles that the hinge must be coaxial with the ulnohumeral joint, and that insertion first requires the position of a provisional pin precisely in the center of rotation of the ulnohumeral joint, which is technically demanding [16]. The procedure requires the precise radiographic determination of the ulnohumeral axis of rotation. Furthermore, the insertion of the pin may make it impossible to position anchors or plates and screw in the distal humerus, and it may require multiple attempts to insert the provisional pin, which may cause overdrilling in the bone. The pin’s insertion in the distal humerus may expose it to risk of infection [4]. An error of only a few millimeters in selecting the axis of rotation could cause changes of several degrees in orientation, determining the early wearing of articular surfaces and instabilities [17]. A study based on the use of an extracorporeal aiming device-based technique demonstrated a more accurate positioning of the hinge than with the conventional pin technique, despite the inability to assess the accuracy of the virtual axis from an antero-posterior view and the difficulty of obtaining a true lateral view [9].

We developed the first auto-centering prototype of a hinged external fixator and then published it in 2015 in collaboration with the Department of Engineering, University of Florence [18].

The idea of developing this external fixation model arose from the need to eliminate the necessity of having to find the center of rotation of the elbow perfectly, in order to reduce positioning attempts and thus possible related joint damage, to reduce exposure to X-rays and operating times.

The auto-centering external fixator is simpler to be applied than the traditional technique as it does not require the position of the pin in the elbow’s center of rotation, nor finding the exact center axis of rotation. These characteristics determine a shorter surgery time and minor exposure to radiation. The error in determining the correct center of rotation of the elbow is like that of the traditional technique. This does not affect the clinical results in our population.

Differing from the first prototype, the newer one has a 30° varus and valgus grade of mobility. Furthermore, while the first type had two different structures based on the left or the right arm, the latter has a unique system for the two sides.

The newest has been simplified in its hinged blocking mechanism and has reduced dimensions, while the first one had a heavy structure. It can also be used as a traditional hinged external fixator with a pin centered on the elbow’s center of rotation. If related to the results of Soubeyrand, the difference in terms of the divergence angle and offset are negligible from the authors’ point of view. The margin of error in positioning the self-centering external fixator in relation to the elbow’s center of rotation is like with the traditional technique, but the advantage of the self-centering technique is that it requires fewer attempts and less X-ray exposure during positioning, as well as avoiding potential complications related to the pin’s positioning, as explained before. This is an initial clinical experiment and further comparative studies need to be developed, but this initial observation is encouraging from our point of view. The limitations of this study are the low number of patients (16 in 10 years) and the short-term follow-up. This paper aims to preliminarily evaluate the results as to whether they are accurate in finding the center of rotation of the elbow. There is no doubt that the authors are interested in publishing their long-term results.

## 5. Conclusions

We think that the auto-centering external fixator could be easier to use, considering that the margin of error in the positioning of the hinge with respect to the axis is comparable with that of the traditional methods, but with respect to them, it shortens the operating time and radiographic exposure and furthermore, does not require starting from the arthrotomic positioning of the guide from the humeral epicondyle.

## Figures and Tables

**Table 1:** Clinical parameters.LCL: lateral collateral ligament, MCUL:medial ulnar collateral ligament, ORIF: open reduction internal fixation.

Patient	Trauma Type	Osteosynthesis and Ligaments	ROM Latest FU	FU (Months)
BC	Simple dislocation	No	135/40	12
CM	Simple dislocation	No	155/0	12
DA	Complex dislocation, with radial head fracture	Radial head replacement, LCL and MCUL repair with anchor	145/20	24
DK	Terrible Triad	Radial head replacement (Coronoid R-M I), LCL repair with anchor	100/5	12
MS	Post-traumatic anchylosis (distal humeral fracture)	Fascia lata interposition arthroplasty, LCL repair with anchor	155/0	96
MM	Terrible Triad	Radial head replacement (Coronoid R-M I), LCL repair with anchor	145/0	24
MR	Post-traumatic anchylosis (Terrible Triad)	Radial head replacement, LCL reconstruction with graft	140/0	24
PA	Post-traumatic anchylosis (Monteggia)	Radial head resection, LCL reconstruction with graft	110/10	24
PR	Post-traumatic terrible triad with subluxation	Radial head replacement, LCL repair with anchor	135/30	48
PM	Post-traumatic terrible triad with subluxation	Radial head replacement, LCL repair with anchor	130/35	24
SA	Terrible Triad	ORIF coronoid and radial head, LCL repair with anchor	135/5	24
BR	Complex dislocation, with radial head fracture	ORIF radial head, LCL repair with anchor	120/15	24
FM	Cronic post-traumatic plus iatrogenic posterolateral instability	LCL repair with anchor	150/10	24
MC	Terrible Triad	ORIF coronoid and radial head, LCL repair with anchor	135/10	12
EF	Terrible Triad	ORIF coronoid, LCL repair with anchor		48
CE	Terrible Triad	ORIF coronoid and radial head replacement, LCL repair with anchor		48

## Data Availability

The raw data supporting the conclusions of this article will be made available by the authors on request.
